# Peripheral administration of SOD1 aggregates does not transmit pathogenic aggregation to the CNS of SOD1 transgenic mice

**DOI:** 10.1186/s40478-021-01211-9

**Published:** 2021-06-22

**Authors:** Isil Keskin, Elaheh Ekhtiari Bidhendi, Matthew Marklund, Peter M. Andersen, Thomas Brännström, Stefan L. Marklund, Ulrika Nordström

**Affiliations:** 1 Department of Medical Biosciences, Pathology, Umeå University, 90185 Umeå, Sweden; 2 Department of Clinical Sciences, Neurosciences, Umeå University, 90185 Umeå, Sweden; 3 Department of Medical Biosciences, Clinical Chemistry, Umeå University, 90185 Umeå, Sweden

**Keywords:** Amyotrophic lateral sclerosis, ALS, Superoxide dismutase 1, SOD1, Protein aggregation, Prion-like, Peripheral administration, Aggregate stability

## Abstract

The deposition of aggregated proteins is a common neuropathological denominator for neurodegenerative disorders. Experimental evidence suggests that disease propagation involves prion-like mechanisms that cause the spreading of template-directed aggregation of specific disease-associated proteins. In transgenic (Tg) mouse models of *superoxide dismutase-1* (*SOD1*)-linked amyotrophic lateral sclerosis (ALS), inoculation of minute amounts of human SOD1 (hSOD1) aggregates into the spinal cord or peripheral nerves induces premature ALS-like disease and template-directed hSOD1 aggregation that spreads along the neuroaxis. This infectious nature of spreading pathogenic aggregates might have implications for the safety of laboratory and medical staff, recipients of donated blood or tissue, or possibly close relatives and caregivers. Here we investigate whether transmission of ALS-like disease is unique to the spinal cord and peripheral nerve inoculations or if hSOD1 aggregation might spread from the periphery into the central nervous system (CNS). We inoculated hSOD1 aggregate seeds into the peritoneal cavity, hindlimb skeletal muscle or spinal cord of adult Tg mice expressing mutant hSOD1. Although we used up to 8000 times higher dose—compared to the lowest dose transmitting disease in spinal cord inoculations—the peripheral inoculations did not transmit seeded aggregation to the CNS or premature ALS-like disease in hSOD1 Tg mice. Nor was any hSOD1 aggregation detected in the liver, kidney, skeletal muscle or sciatic nerve. To explore potential reasons for the lack of disease transmission, we examined the stability of hSOD1 aggregates and found them to be highly vulnerable to both proteases and detergent. Our findings suggest that exposed individuals and personnel handling samples from ALS patients are at low risk of any potential transmission of seeded hSOD1 aggregation.

**Supplementary Information:**

The online version contains supplementary material available at 10.1186/s40478-021-01211-9.

## Introduction

Amyotrophic lateral sclerosis (ALS) is characterized by adult-onset progressive degeneration of upper and lower motor neurons. Symptoms begin focally and then spread contiguously, resulting in progressive paralysis and death from respiratory failure [18]. Previous research suggests that age-related neurodegenerative disorders, including Alzheimer’s disease (AD), Parkinson’s disease (PD), multiple system atrophy (MSA), frontotemporal dementia, and ALS share a common pathogenic mechanism involving a prion-like spread of disease-associated misfolded protein aggregates [4, 11, 23, 43, 48]. Prion-like spread in neurodegenerative diseases may have implications for the safety of relatives, medical staff, and recipients of donated blood or tissue, especially if protein aggregates are able to spread from the periphery into the central nervous system (CNS).

Mutations in the gene encoding superoxide dismutase-1 (SOD1) can cause ALS [53] and are found in 1–9% of all patients, depending on the population [1]. Cytoplasmic neuronal inclusions containing aggregated SOD1 are a hallmark of ALS, both in patients and transgenic (Tg) animal models overexpressing mutant human SOD1 (hSOD1) [32]. A number of studies have provided compelling evidence for a prion-like propagation of hSOD1 aggregation in cell cultures [8, 19, 26, 27, 47, 49]. We previously showed that inoculation of two distinct strains (type A and B) of hSOD1 aggregate seeds, prepared from the spinal cords from terminally ill Tg ALS-model mice into the spinal cords of asymptomatic Tg mice expressing mutant hSOD1, induced spreading of template-directed hSOD1 aggregation and premature fatal ALS-like disease [10, 11]. Furthermore, inoculation of ALS patient-derived hSOD1 aggregate seeds into the spinal cord also induced spreading aggregation, and an aggressive premature motor neuron disease in hSOD1 Tg mice [23]. Moreover, Ayers et al. found that both spinal cord and sciatic nerve inoculations, using homogenates from spinal cords of terminally ill hSOD1 Tg mice, transmit ALS-like disease in mice expressing hSOD1 fused to yellow fluorescent protein [5, 6].

Similar to the infectious isoform of prion protein (PrP^Sc^), aggregates of alpha-synuclein (αSyn), β-amyloid (Aβ), and tau are able to invade the CNS and induce seeded aggregation after peripheral injection in Tg mouse models of PD, MSA and AD [15, 17, 21, 22, 38, 54, 59]. In the case of PD, several studies have further suggested that αSyn aggregation may initially develop in the olfactory bulb, or the gut, and propagate to the CNS via neuroanatomically connected tracts [13, 14, 28, 34, 51].

Here we aimed to investigate whether efficient spread of seeded hSOD1 aggregation and ALS-like symptoms is unique to direct inoculations into the CNS and peripheral nerve, or if peripherally administered hSOD1 aggregates have the potential to spread into the CNS, e.g. via the circulation or neuromuscular junctions. We inoculated large amounts of hSOD1 aggregates into the peritoneal cavity or hindlimb skeletal muscle of adult asymptomatic hSOD1^G85R^ Tg mice. In contrast to the highly pathogenic spinal cord inoculations, peripheral administration of hSOD1 aggregates did not induce spreading aggregation and premature ALS in the recipient mice. To explore potential reasons for the lack of transmission, we examined the stability of strain A hSOD1 aggregates and found them to be highly vulnerable to both proteases and detergent. Our findings suggest a low risk of transmission for potentially exposed individuals and medical staff handling samples from ALS patients carrying *SOD1* mutations.

## Materials and methods

### Mouse husbandry and procedures

In this study, we used hemizygous Tg mice that express hSOD1^G85R^ (line 148) [16] that were backcrossed for more than 30 generations and maintained on the C57BL/6J background. In our colony, the average lifespan of hSOD1^G85R^ mice is 398 ± 42 days (n = 86). There was no significant difference in lifespan between the sexes: females 401 ± 42 days (*n* = 38); males 395 ± 43 days (*n* = 48). Sacrificed non-Tg C57BL/6J mice from the breeding colony were used to make control spinal cord homogenates. All animals were housed under environmentally controlled standard conditions with a 12 h light/dark cycle and free access to food and water. To facilitate free access after the onset of paresis symptoms, a plate with wet food on the floor was provided daily.

### Reagents and chemicals

Reagents and chemicals were obtained from Sigma-Aldrich or Thermo Fisher Scientific unless stated otherwise.

### Preparation of spinal cord homogenates used for inoculations

Homogenates containing strain A hSOD1 aggregates were prepared from the spinal cord and brainstem of terminally ill hSOD1^G85R^ Tg mice (359–446 days old) and control inoculum from a 300-day-old non-Tg C57BL/6 mouse. Whole spinal cords and brainstem from the mice were homogenized in 10 weight volumes (w/v) of phosphate-buffered saline (PBS) using an Ultraturrax apparatus (IKA) for 20 s followed by sonication for 1 min and centrifugation at 1000 g for 10 min at 4 °C. The resulting supernatants were aliquoted and stored at − 80 °C until used for inoculations.

The hSOD1^G85R^ spinal cord homogenate contained 10 µg/ml of total hSOD1 and 1.62 µg/ml detergent-resistant hSOD1 aggregates. The total protein contents of the hSOD1^G85R^ and control homogenates were 2079 µg/ml and 2956 µg/ml, respectively, and were analyzed using the Pierce BCA Protein Assay (Thermo Fisher Scientific).

### Inoculations

Before surgeries, the recipient mice were anesthetized with 5% isofluorane (Baxter) in an induction chamber. The mice were fixed on a small animal stereotactic frame (Kopf Instruments) and anesthesia was maintained with 1.5–2% isofluorane via a facemask. Before starting the surgery, mice were injected subcutaneously with Carprofen (Rimadyl, 5 mg/kg, Pfizer) to relieve pain. The body temperature was monitored and regulated with a TCAT-2LV temperature controller (AgnTho's). After surgeries, physiological saline was injected subcutaneously for rehydration, and mice were placed on a heating blanket for recovery before being returned to their home cage. After the procedure, mice were monitored to ensure full mobility and no signs of impairment. Surgery clips were removed two weeks after surgery.

Intramuscular inoculations (i.m.) of the spinal cord homogenates were performed in ~ 100-day-old asymptomatic hSOD1^G85R^ Tg mice. Mice were anesthetized, and the incision site was shaved and disinfected. A small incision in the skin of the right hindlimb to expose the quadriceps femoris muscle. The needle was inserted ∼ 1 mm deep into the right quadriceps femoris muscle and 10 µl of spinal cord homogenate was injected into the muscle during 3 min. Injections were performed using a 10 µl syringe with a beveled 26-gauge needle (Hamilton). Separate syringes were used for each type of inoculum to prevent any contamination. The syringe was then slowly retracted over 3 min and the skin incision closed with stainless steel clips (Reflex Autoclip System, 10 mm, AgnTho’s).

Spinal cord inoculations were performed as previously described [11, 23]. In summary, the recipient ~ 100-day-old asymptomatic hSOD1^G85R^ Tg mice were anesthetized, and 1 µl of the spinal cord homogenate from the hSOD1^G85R^, or control mice was inoculated into the ventral horn of the left lumbar spinal cord between two vertebrae at the L2–L3 level. Injections were performed using a 5 µl syringe with a 33-gauge needle (Hamilton).

Intraperitoneal inoculations (i.p.) were performed in asymptomatic hSOD1^G85R^ Tg mice that received two injections, each containing 150 µl of the spinal cord homogenate, and administered one week apart. No anesthesia was used on these animals.

### Monitoring of mice

All mice were examined and weighed weekly. No obvious disturbances in bladder control or hindlimb sensory response were observed in any of the inoculated mice. Mice were sacrificed when considered terminally ill. The criterion for the terminal ALS-like disease was defined as advanced paralysis in both hindlimbs or more than 20% loss of body weight. In mice with prominent forelimb symptoms, end-stage criteria also included severe eye infection.

### Excluded mice

Seven i.m. and one spinal cord inoculated hSOD1^G85R^ Tg mice were excluded and not reported in Results, Discussion, Figures or Tables. Four mice that received i.m. inoculation of homogenate from hSOD1^G85R^ Tg spinal cord were euthanized 15, 62, 62, and 112 days after inoculation, respectively, due to non-surgery-related wounds. Three mice inoculated intramuscularly with homogenates from control mice were euthanized 44, 45, and 48 days after inoculation, respectively, due to non-surgery-related wounds. One mouse that received spinal cord inoculation with a 1:3 diluted homogenate from hSOD1^G85R^ Tg spinal cord was euthanized 57 days after inoculation due to wound infection adjacent to the injection site. None of the excluded mice showed any paretic symptoms.

### Tissue handling and immunohistochemistry

The i.p. inoculated mice were sacrificed following a lethal i.p. injection of pentobarbital (60 mg/mL, APL Pharma) and perfused transcardially with PBS. Peripheral tissues (liver, kidney, quadriceps femoris muscle, and sciatic nerve) were collected and snap-frozen in liquid nitrogen. The spinal cord was retrieved by flushing with saline using a syringe inserted caudally to sacral levels and was divided into two sagittal halves, and then into lumbar, thoracic, and cervical segments. One lateral half of each segment was snap-frozen in liquid nitrogen. Frozen tissues were stored at − 80 °C until analysis. The other half of the spinal cord was immersion fixed in formalin for histopathology. The brains were dissected and divided into right and left hemispheres, cerebellum, and brainstem, and processed like the spinal cords. Fixed tissue was paraffin-embedded and 5 µm thick sections were cut with a microtome (Microm HM400) and mounted on glass slides.

Sections from the lumbar spinal cord and brainstem from six i.p. hSOD1^G85R^ homogenate inoculated and six control inoculated mice were processed for immunohistological analysis.

For immunofluorescence staining sections were deparaffinized, rehydrated, and pre-treated with Cell Conditioning 1 (CC1, pH 6, Ventana Medical Systems) solution at 95 °C for 76 min using the Ventana Benchmark Ultra (Ventana Medical Systems). After blocking with 10% (v/v) fetal bovine serum (FBS) in Tris-buffered saline (TBS) containing 0.3% (v/v) Triton X-100 for 1 h at room temperature, sections were incubated with 5% (v/v) FBS in TBS containing 0.1% (v/v) Triton X-100 overnight at 4 °C with primary antibodies. The primary antibodies used were a rabbit anti-hSOD1 antibody raised against a peptide corresponding to amino acids (aa) 131–153 (0.96 μg/ml) and a biotinylated Griffonia Simplicifolia Lectin I (GSL I) isolectin B4 (20 μg/ml, B-1205, Vector Laboratories). Sections were washed with TBS and incubated for 2 h at room temperature with Alexa-Fluor conjugated Streptavidin (1:1000, Invitrogen) and 4′,6-diamidino-2-phenylindole (DAPI; 0.3 μM, Sigma-Aldrich) for nuclear counterstaining. Following washing with TBS, sections were mounted with a Fluorescence Mounting Media (Dako). Images were obtained using an Olympus BX 61 microscope equipped with cellSens Dimension imaging software (cellSens Dimension 1.11, Olympus).

### Quantitative binary epitope-mapping assay

Human SOD1 aggregate contents in the peripheral tissues of i.p. inoculated hSOD1^G85R^ Tg mice were quantified using a binary epitope-mapping (BEM) assay previously described [10]. Tissue homogenates were prepared from the lumbar, thoracic, and cervical spinal cord, brainstem, brain, cerebellum, liver, kidney, quadriceps femoris muscle, and sciatic nerve of i.p. inoculated hSOD1^G85R^ Tg mice. Tissues were homogenized in 25 volumes of ice-cold PBS containing 1.8 mM EDTA, 1 mM DTT, and a Complete EDTA-free protease inhibitor cocktail (Roche Diagnostics) using an Ultraturrax apparatus (IKA) for 20 s followed by sonication 1 min. The resulting crude tissue homogenates were stored at − 80 °C for the subsequent analysis of hSOD1 aggregate contents. The tissue homogenates were further diluted in 20 volumes of PBS containing 1 mM DTT, 1.8 mM EDTA, and 1% (v/v) Nonidet P-40 (NP-40), sonicated for 30 s, and centrifuged at 1000 *g* for 10 min at 4 °C. The resulting supernatants were serially diluted 1:1 in PBS and hSOD1 aggregates were captured on 0.2 µm cellulose acetate filters using a 96-well dot-blot apparatus (Whatman GmbH). The blots were incubated with an anti-hSOD1 primary antibody overnight at 4 °C and developed as described for western blotting. The hSOD1 aggregate content in tissue extracts was determined similar to western blot analysis. The primary antibody used for the quantitative BEM assay was a rabbit antibody raised against a peptide corresponding to aa 57–72 of hSOD1. Of the eight anti-peptide antibodies that cover the hSOD1 sequence and are used in the BEM analysis, this antibody gives the strongest reaction with strain A aggregates. As standard for quantification by BEM assay, we used a frozen aliquot of a spinal cord homogenate from an end-stage hSOD1^G93A^ Tg mouse (set to 1).

### Human SOD1 antibodies

The hSOD1 antibodies used in this study were raised against peptides corresponding to aa 24–39, 57–72, and 131–153 in rabbits as previously described [25, 29], and purified using Protein A-Sepharose (GE Healthcare) followed by Sulfolink gel coupled to the respective target peptides (Thermo Fisher Scientific).

### Strain A aggregate preparations for stability tests

Whole spinal cords from end-stage hSOD1^G85R^ Tg mice were homogenized in 5 volumes of ice-cold PBS containing 1.8 mM EDTA, 0.25 M guanidinium chloride, 2% (v/v) NP-40, and a Complete EDTA-free protease inhibitor cocktail (Roche Diagnostics) using an Ultraturrax apparatus (IKA) for 20 s followed by sonication for 2 min. The homogenate was then diluted with 0.66 volumes of water containing 1% (v/v) NP-40 to achieve physiological ionic strength (0.15 M salt), sonicated for 1 min, and centrifuged at 1000 *g* for 20 min at 4 °C. The supernatant was collected, supplemented with 3% iohexol and transferred to 4 ml UltraClear flexible ultracentrifugation tubes (Thermo Fisher Scientific) containing 0.25 ml (2 mm height) of 75.5% iohexol, followed by a layering of 1.5 ml (10 mm) of 13% iohexol, 1.5 ml (10 mm) of the homogenate containing 3% iohexol, and finally ≈ 0.75 ml PBS to fill up the tubes. The homogenate was then centrifuged through the iohexol density cushion for 2 h at 360,000 *g* at 4 °C and the suspension containing aggregated hSOD1 and other heavy components that sedimented to the 13%/75.5% iohexol interphase was collected. The iohexol was removed by dialysis against PBS (pH 7.0). The resulting preparation, which contained 1.39 µg/ml detergent-resistant hSOD1^G85R^ aggregates and 526 µg/ml total protein, was aliquoted and stored at -80 °C until used for stability analysis of the strain A hSOD1 aggregates.

### Quantification of detergent-resistant hSOD1 aggregates

To determine the content of detergent-resistant hSOD1 aggregates in the homogenate and strain A-aggregate preparation, an aliquot of each was sonicated for 1 min in ice-cold buffer, containing PBS, Complete EDTA-free protease inhibitor cocktail (Roche Diagnostics) and 1% (v/v) NP-40 (for homogenate) or 2% (v/v) NP-40 (for strain A preparation). After sonication, the samples were centrifuged at 337,000 *g* for 3 h at 4 °C and the hSOD1 content in the resulting pellet was analyzed by western blotting. A human hemolysate, calibrated against pure hSOD1, was used as standard for estimations of hSOD1 content.

### Western blotting

Western blots were performed on Any kD Criterion TGX precast gels (BioRad) as previously described [29]. The immunoreactivity was detected using ECL Select reagent (GE Healthcare), recorded on a ChemiDoc Touch Imaging System (BioRad), and analyzed using Image Lab software (BioRad). The primary antibody used for western blot experiments was a rabbit anti-hSOD1 antibody raised against a peptide corresponding to aa 24–39 (1.7 μg/ml). This antibody is human-specific and does not detect murine SOD1.

### Stability tests of strain A aggregates

The hSOD1 aggregate preparation was vortexed for 2 min and then 10 µl aliquots were incubated with or without different concentrations of trypsin (10, 100 and 1000 µg/ml; Sigma-Aldrich); proteinase K (10, 50 and 250 µg/ml; Thermo Fisher Scientific); sodium dodecyl sulfate (SDS; 0.3, 1, 3 and 10 g/L; Sigma-Aldrich); or glycochenodeoxycholic acid (GCDCA; 8 mM; Sigma-Aldrich) in a total volume of 50 µl of PBS for different time intervals (0, 0.5, 1, 2, 4 and 6 h) at 37 °C using a shaker (IKA). GCDCA incubation was performed in the presence of the Complete EDTA-free protease inhibitor cocktail (Roche Diagnostics). Proteolysis with proteinase K was terminated by the addition of phenylmethylsulphonylflouride (PMSF; 20 and 100 mM; Sigma-Aldrich). After completed incubation, each sample reaction was immediately attenuated by the addition of 500 µl of water as a diluent, snap-frozen in liquid nitrogen and stored at − 80 °C. Frozen samples were thawed in a water bath at 25 °C for 2 min and then centrifuged at 25,000 *g* for 30 min at 4 °C and the supernatants were transferred to new tubes. The pellets were washed by suspension in 1 ml PBS and centrifuged again at 25,000 *g* for 30 min at 4 °C. The amount of hSOD1 in both supernatant and pellet was determined by western blotting.

### Statistical analyses

Statistical analyses were performed using Prism version 6.00 (GraphPad). To test for statistical significance between two groups, Mann–Whitney *U* test was used. Alpha ≤ 0.05 was used as the cut-off for significance. All values are given as mean ± SD.

## Results

### Peripheral inoculations of transgenic homogenates do not induce premature ALS

**Fig. 1:**
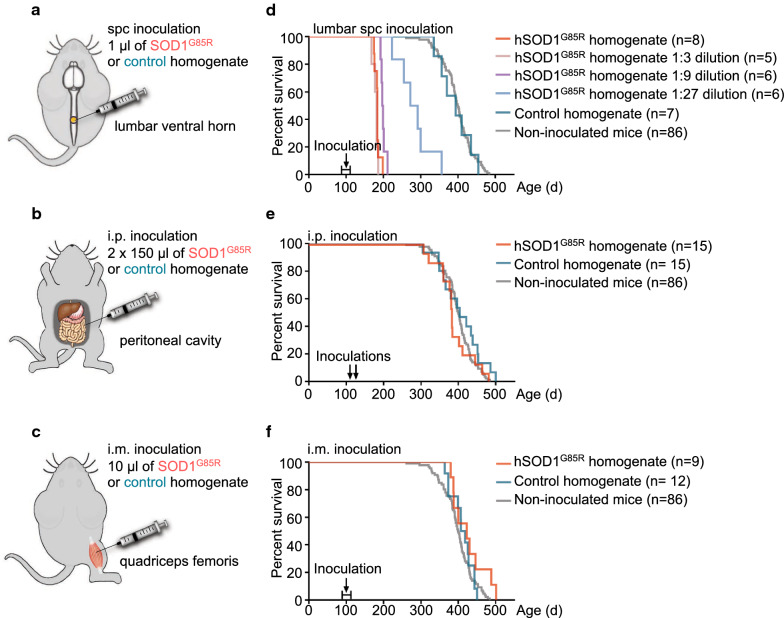
Peripheral inoculations of spinal cord homogenate from paralytic end-stage hSOD1^G85R^ mice do not induce premature ALS in Tg mice. **a**–**c** Experimental overview: ~ 100-day-old pre-symptomatic hSOD1^G85R^ Tg ALS-model mice were inoculated with spinal cord tissue homogenates from either end-stage paralytic hSOD1^G85R^ Tg mice or control non-Tg C57BL/6 mice. We administrated the homogenates **a** into the spinal cord (spc) (single inoculation into the left ventral horn of the lumbar spinal cord, 1 µl), **b** i.p. (two inoculations into the peritoneal cavity, 150 µl each, 1 week apart), and **c** i.m. (single inoculation into the right quadriceps femoris muscle, 10 µl). **d–f** Kaplan–Meier plots show the survival of the spinal cord homogenate inoculated hSOD1^G85R^ Tg mice. As a reference, the plots contain survival data for non-inoculated hSOD1^G85R^ Tg mice. **d** Concentration-dependent induction of premature fatal motor neuron disease after intraspinal inoculation of the Tg homogenate. Survival of hSOD1^G85R^ Tg mice after spinal cord inoculation of an undiluted 10% homogenate from end-stage hSOD1^G85R^ Tg spinal cord and 1:3, 1:9 and 1:27 dilutions, compared to inoculation with control extract

We performed seed inoculations into the peritoneal cavity (i.p.) and quadriceps femoris muscle (i.m.) and compared with the effect of inoculations into the lumbar spinal cord (Fig. 1, Additional file 1: Table S1). The recipients were asymptomatic 100-day-old hSOD1^G85R^ Tg mice, a well-characterized ALS mouse model, which has the advantage of a long symptom-free period followed by a late middle-age onset of aggregation and paresis [11, 23].

As seeds, we used whole spinal cord homogenate preparations from end-stage hSOD1^G85R^ Tg mice and non-Tg C57BL/6 control mice. The hSOD1^G85R^ spinal cord homogenate, which contained ~ 1.7 ng/µl of strain A hSOD1 aggregates, was found to be highly active. Inoculations into the spinal cord using 1 µl of undiluted homogenate, as well as three- and ninefold dilutions, resulted in close to identical shortened survivals. Even a 27-fold diluted homogenate induced seeded pathogenic aggregation and premature motor neuron disease that resulted in a significant shortening of the lifespan of inoculated mice (Fig. 1d, Additional file 1: Table S1).

In contrast, i.p. inoculation of 300 µl of the homogenates, which is more than 8000 times the smallest effective dose used in spinal cord inoculations, did not induce premature paralysis in the hSOD1^G85R^ Tg mice (Fig. 1e). Likewise, inoculations of 10 µl of the homogenate into the quadriceps femoris muscle failed to transmit seeding of premature ALS-like disease (Fig. 1f). There were no differences in survival between the mice that received i.p. or i.m. inoculations of hSOD1^G85R^ Tg homogenates compared to recipients of non-Tg control spinal cord homogenates (Fig. 1e, f and Additional file 1: Table S1).

### Intraperitoneal administration of transgenic homogenate does not induce hSOD1 aggregation in the CNS or peripheral tissues

**Fig. 2:**
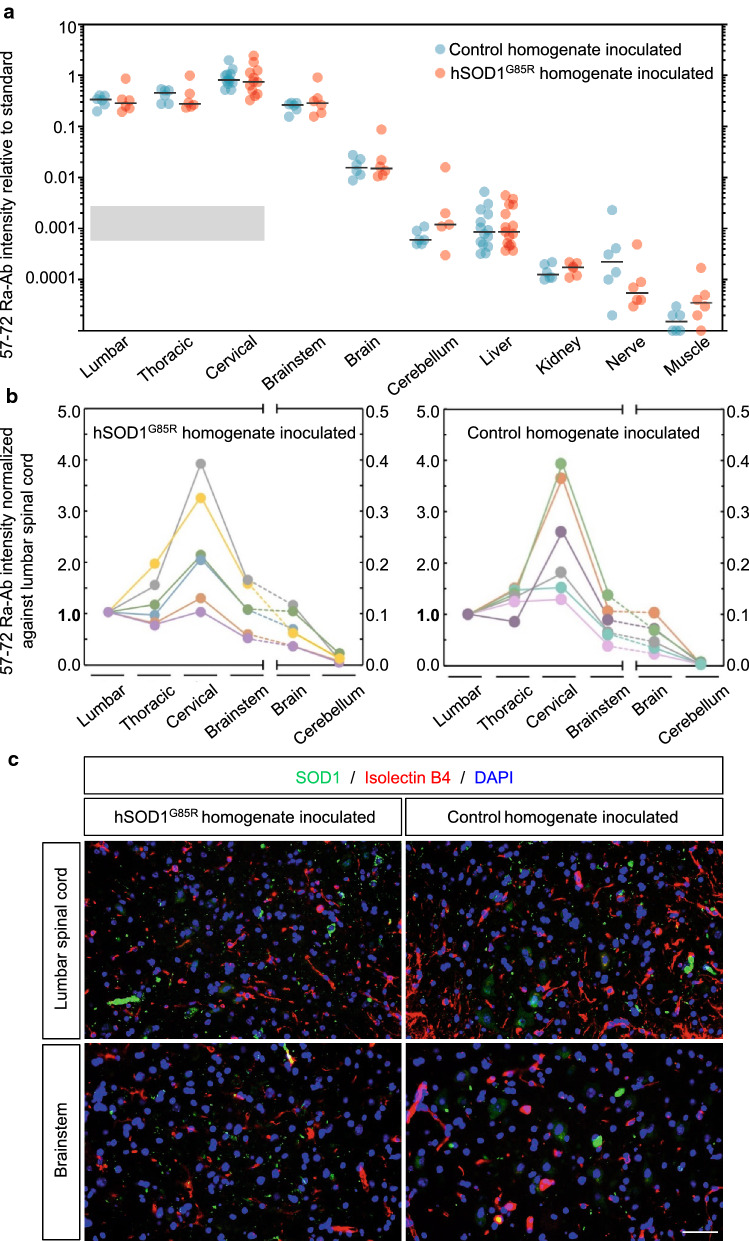
Intraperitoneal administration of hSOD1 aggregate seeds does not induce hSOD1 aggregation in the CNS or peripheral tissues. **a** and **b** Plots depict hSOD1 aggregates detected in the CNS (lumbar, thoracic, and cervical spinal cord, brainstem, brain and cerebellum) and peripheral organs (liver, kidney, sciatic nerve and quadriceps femoris muscle) of hSOD1^G85R^ Tg mice that were inoculated with hSOD1^G85R^ Tg or control spinal cord homogenates into the peritoneal cavity. Amounts of hSOD1 aggregates in tissue homogenates were analyzed with a binary epitope mapping assay using the hSOD1 57–72 Ra-Ab with an end-stage hSOD1^G93A^ spinal cord homogenate used as standard (set to 1). **a** Black line indicates the median value. The shaded area indicates the range of background reactivity to spinal cord homogenates from non-Tg and SOD1-knockout mouse controls (mean ± 2 SD) (n = 8) [10]. Note that the antibody likely would show different background reactivities to dot-blotted homogenates of other tissues from controls. **b** Distribution of hSOD1 aggregates along the neuraxis in individual end-stage Tg mice. The amount of filtertrapped aggregates from different segments was normalized against the levels from the lumbar spinal cord. Note the altered scale used to visualize reactivity to brain and cerebellum homogenates. The results for individual mice are presented in different colors to improve distinction. **c** Immunohistopathological analysis of tissue sections from the lumbar spinal cord and brainstem of end-stage i.p. inoculated hSOD1^G85R^ Tg mice. Tissue sections from the lumbar spinal cord and brainstem were stained using the hSOD1 131–153 Ra-Ab (green), isolectin B4 (red) as a marker for endothelial cells and DAPI (blue). Aggregated hSOD1 was similarly distributed around blood vessels in the lumbar spinal cord and brainstem in the end-stage mice inoculated with either hSOD1^G85R^ Tg spinal cord homogenate or control homogenate. Scale bar represents 50 μm

Although SOD1 is ubiquitously expressed, end-stage paralytic hSOD1^G85R^ Tg mice that accumulate a substantial hSOD1 aggregation in the spinal cord do not develop detectable hSOD1 aggregations in peripheral organs [10]. To determine whether the copious i.p. homogenate inoculations induced aggregation of the ubiquitously expressed hSOD1^G85R^ in organs non-secluded by the blood–brain barrier, we analyzed hSOD1 aggregation in the liver and kidney, which both express higher levels of hSOD1^G85R^ protein than spinal cord tissue [16]. Skeletal muscle and sciatic nerve were also analyzed (Fig. 2a, Additional file 1: Table S2). We found no evidence for transmission of hSOD1 aggregation in peripheral tissues. The reactivity of the antibody in liver homogenates was comparable to the background reactivity with non-Tg control, or SOD1-knockout spinal cord homogenates (Fig. 2a), and about 1000 times lower than end-stage hSOD1^G85R^ Tg spinal cord homogenates [11, 23]. The reactivity was even lower in skeletal muscle, kidney and peripheral nerve. There were no significant differences between mice inoculated with homogenates from hSOD1^G85R^ Tg and the non-Tg control mice (Fig. 2a, Additional file 1: Table S2).

Even though there was no effect on the survival of the i.p. inoculated mice, we determined the distribution of hSOD1 aggregation along the neuraxis (Fig. 2b). We found no significant differences in distribution of hSOD1 aggregation in the neuraxis between mice inoculated with homogenates from hSOD1^G85R^ Tg and non-Tg control mice. The highest levels of hSOD1 aggregates were detected in the cervical spinal cord in both i.p. inoculated groups (Fig. 2a, b, Additional file 1: Table S2).

It has previously been shown that peripheral administration of Aβ results in distribution of Aβ aggregation close to blood vessels in the brain which is different from Aβ aggregation induced by intracerebral inoculations [22]. To further explore the distribution of hSOD1 aggregation, we performed an immunohistopathological analysis of tissue sections from the lumbar spinal cord and brainstem of end-stage i.p. inoculated mice. Human SOD1 aggregates around the blood vessels in the ventral horn of the lumbar spinal cord and brainstem were similarly distributed in end-stage hSOD1^G85R^ Tg mice i.p. inoculated with extracts from the hSOD1^G85R^ Tg and control mice (Fig. 2c).

### Stability of spinal cord-derived hSOD1^G85R^ aggregates

We found that large amounts of peripherally administrated hSOD1 aggregates did not induce premature ALS in hSOD1^G85R^ Tg mice or aggregation in the peripheral organs (Figs. 1, 2a and Additional file 1: Tables S1, S2). To explore the physical stability of the aggregates as a contributing factor to the lack of disease transmission, we examined the resistance of hSOD1 aggregates to proteases and detergents. This evaluation is also of importance for the risk assessment of handling potentially hazardous materials from patients or models of pathogenic aggregation.

**Fig. 3:**
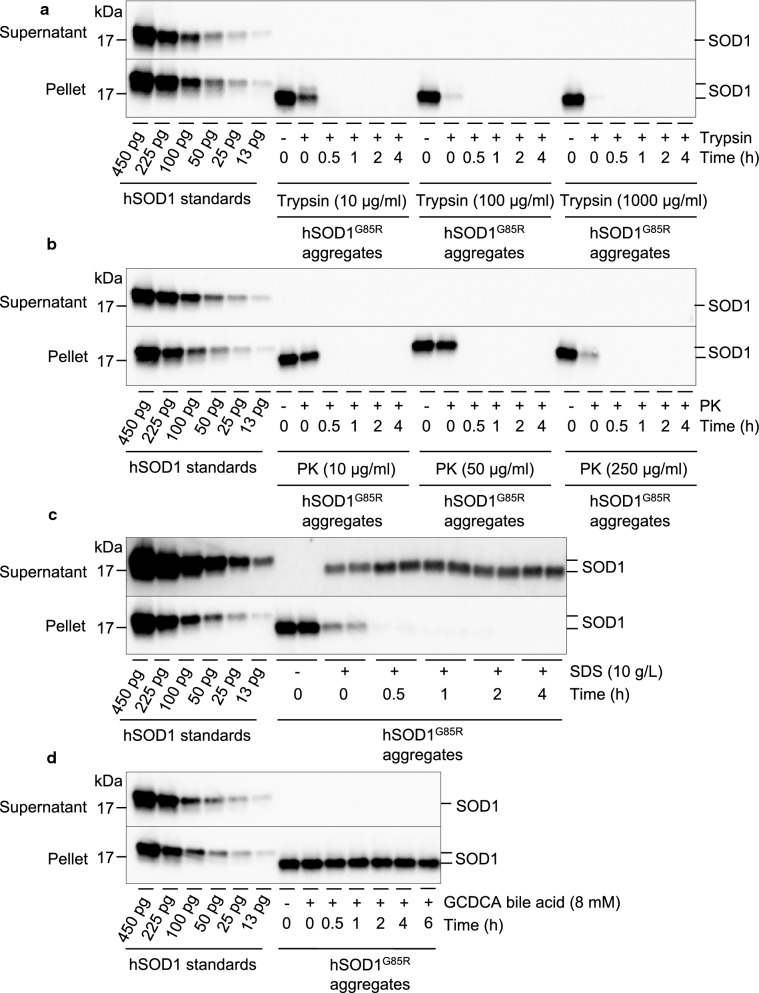
Stability of spinal cord-derived hSOD1^G85R^ aggregates. Human SOD1^G85R^ aggregates were incubated either with or without **a** trypsin (10, 100 and 1000 µg/ml), **b** proteinase K (PK; 10, 50 and 250 µg/ml), **c** sodium dodecyl sulfate (SDS; 10 g/L) or **d** glycochenodeoxycholic acid (GCDCA; 8 mM) for different time intervals (0, 0.5, 1, 2, 4 and 6 h) at 37 °C. **a–d** Amounts of hSOD1, in both supernatant and insoluble fraction, were determined by western blotting. A human-specific SOD1 24–39 peptide antibody was used, and the reactivities was compared to a wild-type hSOD1 standard. Note that the hSOD1 standard has a lower electrophoretic mobility than the hSOD1^G85R^ mutant

Natively folded wild-type SOD1 is an extremely stable protein that is resistant to high concentrations of guanidinium chloride, urea, proteases, and detergents, whereas mutant SOD1 variants with a structural loosening of the native fold and misfolded SOD1 exhibit variable sensitivities [3, 7, 9, 24, 26, 33, 37, 40, 41, 50, 55]. To assess the resistance of strain A hSOD1 aggregates, we exposed a spinal cord-derived hSOD1^G85R^ aggregate preparation to different concentrations of proteases and detergents and incubated the samples for different time intervals at 37 °C. Samples were centrifuged at 25,000 *g* before hSOD1 content in both supernatant and insoluble pellet fractions were analyzed by western blotting (Fig. 3, Additional file 1: Figure S1).

Both trypsin and proteinase K efficiently degraded the aggregates and no hSOD1 was detected in the insoluble fraction after 30 min of incubation (Fig. 3a, b). We did not detect hSOD1 in the soluble fraction at any time point. Thus, hSOD1 aggregates are highly sensitive to protease digestion.

Next, we determined the solubility of hSOD1^G85R^ aggregates following incubation with different concentrations of sodium dodecyl sulfate (SDS) (Fig. 3c, Additional file 1: Figure S1). We found that the mutant SOD1 aggregates were completely dissolved by 10 g/L of SDS for 1 h at 37 °C and appeared in the supernatant fraction (Fig. 3c). Thus, strain A SOD1 aggregates were highly susceptible to SDS. However, SOD1 aggregates were resistant to treatment with a high concentration of another anionic detergent, glycochenodeoxycholic acid, which is the principal bile acid in the gut (Fig. 3d).

## Discussion

Multiple studies have demonstrated that peripherally administrated disease-associated proteins linked to other neurodegenerative diseases have a high potency to transmit aggregation to the CNS. PD- and MSA-associated αSyn aggregates, and AD-associated Aβ- and Tau aggregates, can transmit and spread disease-associated aggregation into the CNS via several different routes [15, 17, 20–22, 31, 38, 39, 44, 46, 54, 59]. Nonetheless, both higher concentrations and longer incubation periods were required for peripherally delivered seeds compared to intracerebral inoculations [21, 22, 54]. In contrast, our results suggest that i.p. or i.m injections of large amounts of hSOD1 aggregates with prion-like properties do not transmit spreading pathogenic aggregation or premature fatal ALS-like disease in the hSOD1^G85R^ Tg model, which is highly susceptible to seeding of template-directed aggregation. The hSOD1^G85R^ spinal cord homogenate used as seeds was highly potent and even a 27-fold dilution readily induced ALS-like symptoms following spinal cord inoculations (Fig. 1d, Additional file 1: Table S1). Still, administration of 270 times more spinal cord homogenate via the i.m route or an 8000 times larger amount via the i.p. route did not induce premature fatal ALS-like disease (Fig. 1e, f, Additional file 1: Table S1). Moreover, there were no differences in distribution of aggregation pathology along the neuraxis in the hSOD1 aggregate inoculated, versus control inoculated mice. Thus, our current data suggest that the potential for transmission of SOD1-prion activity from the periphery to the CNS is very low.

SOD1 is ubiquitously expressed, and the levels of SOD1 in the liver and kidney are six and three times higher, respectively, than in the CNS [29, 30, 42]. We, therefore, investigated whether high amounts of peripherally administrated hSOD1 aggregates would accumulate and possibly induce aggregation in tissues outside the CNS. Our highly sensitive BEM assay showed that there were no significant differences in aggregate load when comparing liver, kidney, skeletal muscle, or sciatic nerves from end-stage Tg mice, inoculated i.p. with control or hSOD1 aggregate containing homogenates (Fig. 2a, Additional file 1: Table S2). This finding is in line with previous studies in AD where no Aβ deposition was found in peripheral organs of end-stage transgenic mice following i.p. inoculations [22]. However, the amyloid precursor protein, as well as Aβ-peptides, are generally only sparsely expressed outside of the CNS [2, 52].

We showed that strain A SOD1 aggregates in preparations from hSOD1^G85R^ Tg mouse spinal cords are highly sensitive to degradation by proteases. These results corroborate with in cell-, and in vitro studies by Grad et al. which demonstrated that mutant SOD1 species, capable of transmitting misfolding to natively folded wild-type SOD1, are sensitive to protease treatment [26]. We further show that strain A SOD1 aggregates are efficiently degraded by exposure to SDS, which is a constituent of commonly used household and laboratory detergents (Fig. 3a–c). However, exposure to another anionic detergent, glycochenodeoxycholic acid, did not dissolve hSOD1 aggregates (Fig. 3d). Possibly the long flexible non-polar alkane chain in SDS interacts with and disrupts the hSOD1 fibril core more efficiently than the bulky partly polar steroid nucleus in the bile acid. Future studies of potential spreading via the gastro-intestinal route may shed light on possible uptake and spread via the enteric nervous system. However, although the aggregates were resistant to bile acid, the high sensitivity to proteases suggests that SOD1 aggregates should be efficiently degraded by the abundant proteases in the gastrointestinal tract, if ingested.

In contrast, like the highly infectious PrP^Sc^, aggregated αSyn and Aβ are remarkably stable and resistant to inactivation. Moreover, both Aβ and αSyn aggregates extracted from the brain tissue of AD, PD, or MSA patients and Tg mice are highly robust and retain their seeding activity after exposure to both detergents and proteinase K [12, 35, 36, 45, 56–59]. Hence, inferior physical stability and low resistance to proteases may be contributing factors to why peripherally administrated hSOD1 aggregates did not transmit seeded aggregation to the CNS.

## Conclusions

While inoculation of minute amounts of SOD1 aggregates with prion-like properties into the CNS readily induces spreading aggregation and premature fatal motor neuron disease, peripheral administration of large amounts did not induce disease in the hSOD1 Tg mice. Moreover, although SOD1 is ubiquitously expressed, no SOD1 aggregation was detected in peripheral tissues. These findings indicate that any potential risk for transmission of pathogenic SOD1 aggregation in exposed personnel handling samples from ALS patients or transgenic disease models should be considered low. However, precautionary safety measures should still be taken, especially while handling samples containing elevated concentrations of hSOD1 aggregates.

To this end, there is a need for guidelines for handling procedures and decontamination of laboratory material and surfaces, medical devices, and surgical instruments. Our results indicate that solutions of common commercially available detergents, containing > 1% SDS, efficiently degrade mutant hSOD1 aggregates.

## Supplementary Information

**Additional file 1**. Disease and survival data, SOD1 aggregate levels in tissue samples, and detergent resistance of hSOD1 aggregates.

## Data Availability

All data generated or analysed during this study are included in this published article and its supplementary information files.

## Abbreviations

ALS: amyotrophic lateral sclerosis
hSOD1: human SOD1
SOD1: superoxide dismutase-1
