# Supplementary material for: Peripheral administration of SOD1 aggregates does not transmit pathogenic aggregation to the CNS of SOD1 transgenic mice
Source: Acta Neuropathol Commun. 2021 Jun 22;9:111. doi: 10.1186/s40478-021-01211-9 (PMC8220797; doi:10.1186/s40478-021-01211-9)
Supplement: Supplementary file 1 — Additional file 1. Disease and survival data, SOD1 aggregate levels in tissue samples, and detergent resistance of hSOD1 aggregates. [file 40478_2021_1211_MOESM1_ESM.pdf]

Supplementary Information

Peripheral administration of SOD1 aggregates does not transmit pathogenic aggregation to the CNS of SOD1 transgenic mice

Isil Keskin[1], Elaheh Ekhtiari Bidhendi[2], Matthew Marklund[1], Peter M. Andersen[2], Thomas Brännström[1], Stefan L. Marklund[3], Ulrika Nordström[2,*]

[1] Department of Medical Biosciences Pathology, Umeå University, Umeå 90185, Sweden.
[2] Department of Clinical Sciences, Neurosciences, Umeå University, Umeå 90185, Sweden.
[3] Department of Medical Biosciences, Clinical Chemistry, Umeå University, Umeå 90185, Sweden.

* To whom correspondence should be addressed.
Email: ulrika.nordstrom@umu.se
Tel: +46 - 73 739 6695

Table S1 Characteristics of the motor neuron disease in inoculated mice

	Intraperitoneal inoculation				Intramuscular inoculation			
	hSOD1^{G85R}		Control		hSOD1^{G85R}		Control	
	n		*n*		*n*		*n*	
Age at inoculation (days)	15	(119 and 126) ± 14^{ns}	15	(114 and 121) ± 7	9	105 ± 5^{ns}	12	106 ± 1
Time to symptom onset (days)	15	257 ± 50^{ns}	15	272 ± 51	9	303 ± 41^{ns}	12	285 ± 29
Time to fatal disease (days)	15	270 ± 51^{ns}	15	292 ± 57	9	322 ± 46^{ns}	12	304 ± 29
Age at fatal disease stage (days)	15	389 ± 47^{ns}	15	406 ± 55	9	427 ± 44^{ns}	12	411 ± 29
Weight loss from maximum weight (%)	15	31 ± 7^{ns}	15	34 ± 8	9	35 ± 6^{ns}	12	36 ± 9

	Intraspinal inoculation									
	hSOD1^{G85R}		hSOD1^{G85R} 1:3 dilution		hSOD1^{G85R} 1:9 dilution		hSOD1^{G85R} 1:27 dilution		Control	
	n		*n*		*n*		*n*		*n*	
Age at inoculation (days)	8	108 ± 3**	5	103 ± 7^{ns}	6	109± 0***	6	104 ± 1^{ns}	7	103 ± 1
Time to symptom onset (days)	8	51± 9***	5	55± 9**	6	57± 11**	6	99± 18**	7	258 ± 24
Time to fatal disease (days)	8	76 ± 6***	5	76 ± 5**	6	90 ± 7**	6	179 ± 46**	7	291 ± 43
Age at fatal disease stage (days)	8	184 ± 7***	5	179 ± 7**	6	199 ± 7**	6	283 ± 45**	7	394 ± 43
Weight loss from maximum weight (%)	8	26 ± 5**	5	26 ± 7*	6	27 ± 5*	6	25 ± 11^{ns}	7	35 ± 7

The table shows data for mice predetermined for lifespan analysis. Data are expressed as the mean ± SD, *$p \leq 0.05$; **$p \leq 0.01$; ***$p \leq 0.001$, analyzed by Mann–Whitney U test comparing the hSOD1^{G85R} and control homogenates within each inoculation group. *ns* not significant.

Table S2 Human SOD1 aggregate levels in the CNS and peripheral organs of the intraperitoneally inoculated hSOD1^{G85R} Tg mice

Inoculated homogenate	Lumbar (n= 6)	Thoracic (n= 6)	Cervical (n= 12)	Brainstem (n= 6)	Brain (n= 6)	Cerebellum (n= 5 or 6)
Control	0.324 ± 0.079	0.418 ± 0.117	0.940 ± 0.405	0.247 ± 0.052	0.017 ± 0.007	0.00070 ± 0.00024
hSOD1^{G85R}	0.366 ± 0.251^{ns}	0.412 ± 0.295^{ns}	0.945 ± 0.637^{ns}	0.363 ± 0.278^{ns}	0.027 ± 0.030^{ns}	0.00410 ± 0.00662^{ns}

Inoculated homogenate	Liver (n= 15)	Kidney (n= 6)	Skeletal muscle (n= 6)	Peripheral nerve (n= 6)
Control	0.00139 ± 0.00138	0.00015 ± 0.00005	0.00002 ± 0.00001	0.00055 ± 0.00087
hSOD1^{G85R}	0.00153 ± 0.00137^{ns}	0.00017 ± 0.00005^{ns}	0.00005 ± 0.00006^{ns}	0.00013 ± 0.00018^{ns}

Table shows the amount of hSOD1 aggregates detected in the CNS (lumbar, thoracic, and cervical spinal cord, brainstem, brain and cerebellum) and peripheral organs (liver, kidney, sciatic nerve and quadriceps femoris muscle) of the hSOD^{G85R} Tg mice that were intraperitoneally inoculated with spinal cord homogenates from hSOD^{G85R} Tg or control mice (related to Fig. 2a). Amount of hSOD1 aggregates analysed with binary epitope mapping using the 57-72 Ra-Ab with an end-stage hSOD1^{G93A} spinal cord homogenate used as standard. All data are presented as mean ± SD and significances analyzed by Mann–Whitney U test comparing the hSOD1^{G85R} and control homogenates within each tissue, *ns* not significant.

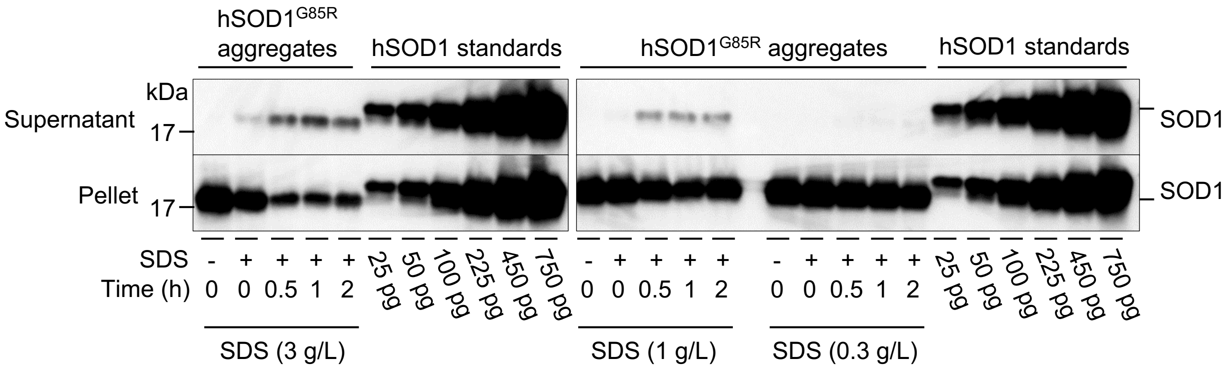

Figure S1 Stability of spinal cord-derived hSOD1^{G85R} aggregates (related to Fig. 3c).

SOD1 aggregates derived from hSOD1^{G85R} Tg mice spinal cords were incubated with or without sodium dodecyl sulfate (SDS; 0.3, 1 and 3 g/L) for different time intervals (0, 0.5, 1 and 2 h) at 37°C while shaking. The amount of hSOD1 was determined in the supernatant and pellet by western blotting using the 24-39 Ra-Ab, by comparison to a hSOD1 standard. Note that wild-type hSOD1 has a lower electrophoretic mobility than hSOD1^{G85R}.
